# TPP riboswitch-dependent regulation of an ancient thiamin transporter in *Candida*

**DOI:** 10.1371/journal.pgen.1007429

**Published:** 2018-05-31

**Authors:** Paul D. Donovan, Linda M. Holland, Lisa Lombardi, Aisling Y. Coughlan, Desmond G. Higgins, Kenneth H. Wolfe, Geraldine Butler

**Affiliations:** 1 School of Biomedical and Biomolecular Science and UCD Conway Institute of Biomolecular and Biomedical Research, Conway Institute, University College Dublin, Belfield, Dublin 4, Ireland; 2 School of Medicine and UCD Conway Institute of Biomolecular and Biomedical Research, University College Dublin, Belfield, Dublin 4, Ireland; Carnegie Mellon University, UNITED STATES

## Abstract

Riboswitches are non-coding RNA molecules that regulate gene expression by binding to specific ligands. They are primarily found in bacteria. However, one riboswitch type, the thiamin pyrophosphate (TPP) riboswitch, has also been described in some plants, marine protists and fungi. We find that riboswitches are widespread in the budding yeasts (Saccharomycotina), and they are most common in homologs of *DUR31*, originally described as a spermidine transporter. We show that *DUR31* (an ortholog of *N*. *crassa* gene *NCU01977*) encodes a thiamin transporter in *Candida* species. Using an RFP/riboswitch expression system, we show that the functional elements of the riboswitch are contained within the native intron of *DUR31* from *Candida parapsilosis*, and that the riboswitch regulates splicing in a thiamin-dependent manner when RFP is constitutively expressed. The *DUR31* gene has been lost from *Saccharomyces*, and may have been displaced by an alternative thiamin transporter. TPP riboswitches are also present in other putative transporters in yeasts and filamentous fungi. However, they are rare in thiamin biosynthesis genes *THI4* and *THI5* in the Saccharomycotina, and have been lost from all genes in the sequenced species in the family Saccharomycetaceae, including *S*. *cerevisiae*.

## Author summary

Thiamin, or Vitamin B1, is an essential requirement in all living organisms because it is a co-factor for many enzymes in metabolism. Unlike animals, many yeasts can synthesize thiamin, or they can import it from the environment. Expression of thiamin biosynthesis genes and of thiamin transporters is strictly regulated in response to the presence of thiamin. In many filamentous fungi, expression of thiamin biosynthesis genes is regulated by TPP riboswitches, RNA regulatory elements that are located within messenger RNA. TPP riboswitches are rare in yeasts. However, we find that TPP riboswitches are conserved in an ancient thiamin transporter, found in filamentous fungi, yeasts and other related organisms. There appears to be a high turnover of thiamin transporters in fungi, and there has been a gradual loss of TPP riboswitches in yeasts.

## Introduction

Riboswitches are RNA regulatory elements that are located within messenger RNA, and control gene expression [1]. The most common classes bind to small molecule ligands, ranging from coenzymes, *S*-adenosylmethionine (SAM) and amino acids to metal ions [2]. Other classes respond to temperature [3], pH [4], and tRNA binding [5]. Binding of the ligand or changing the temperature or pH disrupts the secondary structure of the riboswitch.

Riboswitches are best described in bacteria, where over 20 ligands have been identified [2, 6]. Bacterial riboswitches are usually located in 5’ UTR regions, and control initiation of translation or premature termination of transcription. Ligand binding introduces a structural change that prevents access to the ribosome binding site, or promotes the formation of an intrinsic transcriptional terminator. In eukaryotes only one type of riboswitch has been identified, which binds thiamin pyrophosphate (TPP, a derivative of thiamin). TPP riboswitches regulate expression of thiamin synthesis genes in algae and marine phytoplankton [7], plants [8, 9] and filamentous fungi [10, 11], and probably in oomycetes [12]. Eukaryotic riboswitches are often located within introns, and they function by regulating splicing.

Thiamin is an enzyme cofactor that can be imported into the cell, or can be synthesized from 5-(2-hydroxyethyl)-4-methylthiazole phosphate (HET-P) and 4-amino-5-hydroxymethyl-2-methylpyrimidine diphosphate (HMP-PP). Thiamin is converted to TPP through the activity of thiamin pyrophosphokinase (*THI80* in yeast [13]). The filamentous fungus *Neurospora crassa* has three riboswitches, two of which are in introns in the 5’ UTRs of the thiamin synthesis genes *THI4* (*NCU06110*) and *THI5* (also known as *NMT1* or *NCU09345*) [10, 11]. Splicing of these introns uses at least two 5’ donor splice sites, depending on the environmental conditions. In *THI5*, in the absence of TPP, a small region of the riboswitch base pairs with a complementary sequence surrounding the second donor splice site, preventing access to the splicing machinery [10]. Splicing therefore occurs at the first donor splice site, removing the entire intron, and facilitating translation of a functional protein. In the presence of TPP, the riboswitch adopts a different structure, which disrupts its interaction with the second donor splice site. Splicing at the second site is now favored, and part of the intron is retained in the mRNA. Small upstream open reading frames (uORFs) in the retained intron compete with the main ORF for translation. A similar process occurs in *THI4*, which has a slightly more complex intron structure.

Expression of *N*. *crassa* gene *NCU01977* is regulated by a third TPP riboswitch, but somewhat differently to *THI4* and *THI5* [11]. The riboswitch in *NCU01977* is in an intron within the coding sequence, not in the 5’ UTR. The intron has several potential 5’ splice sites, and only splicing at the first site produces a fully functional protein. Splicing at other sites results in the introduction of premature stop codons. In the absence of TPP, a long-range interaction between the riboswitch and a region adjacent to the first splice site increases the use of this site, possibly by looping out the intermediate donor splice sites [11]. When thiamin is present, splicing occurs at the downstream donor sites and translation stops prematurely. The logic of the switch remains the same—thiamin or TPP represses translation of *NCU01977—*but the mechanism is different to the thiamin biosynthesis genes. *NCU01977* encodes a putative transporter. Genes with similar domains in other filamentous fungi, and in phytoplankton, also contain TPP riboswitches [7]. In marine algae, it has been hypothesized that *NCU01977* orthologs may transport thiamin or thiamin intermediates such as HMP or AmMP, but experimental evidence is lacking [7, 14].

In some algae, it has been predicted that riboswitches regulate expression of thiamin biosynthesis genes by base pairing to the branch point of the intron in the presence of TPP, preventing splicing [15]. The resulting messenger RNA contains premature stop codons. In plants, a 3’ processing site in the mRNA is removed by riboswitch controlled alternative splicing in the presence of TPP, resulting in transcripts with reduced stability [9]. Thiamin or TPP therefore represses translation of the target genes in filamentous fungi, algae and plants by controlling alternative splicing, but through many different mechanisms.

Until recently, TPP riboswitches have generally been assumed to be absent from budding yeasts (Saccharomycotina), although a few have been predicted bioinformatically [12, 16–18]. We find that TPP riboswitches are common in *DUR31* genes in the Saccharomycotina, and that splicing of this gene in *Candida parapsilosis* is regulated by thiamin. We show for the first time that *DUR31*, which is an ortholog of *Neurospora crassa NCU01977*, encodes a thiamin transporter.

A small number of yeast species retain riboswitches in thiamin biosynthesis genes, but all TPP riboswitches have been lost from all sequenced species in the family Saccharomycetaceae, including *S*. *cerevisiae*. Riboswitch-mediated regulation of thiamin transport is therefore strongly conserved throughout fungi, including yeasts, as well as in algae and plants.

## Results

### TPP riboswitches are present in *Candida* species

**Fig 1:**
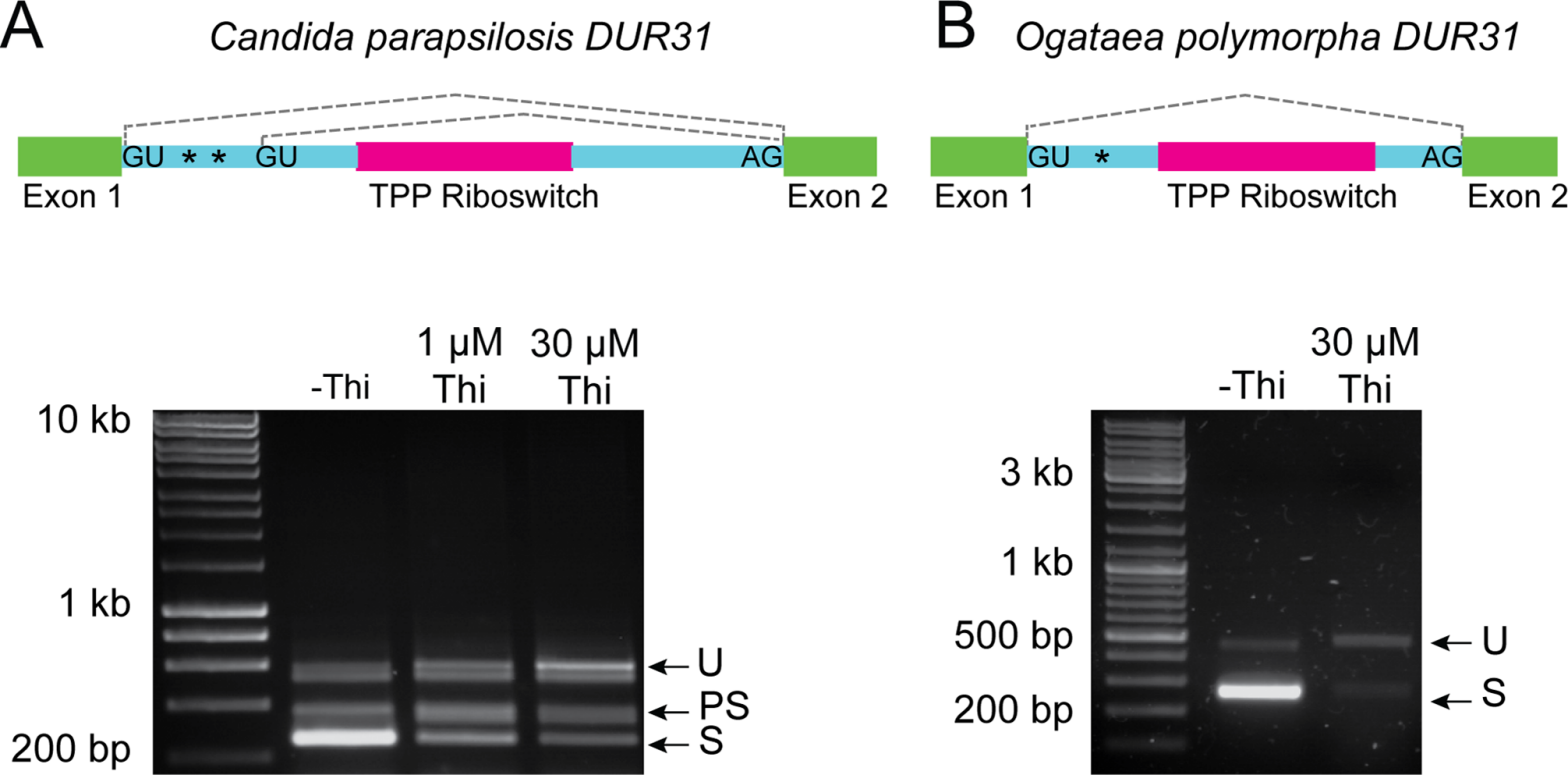
Thiamin regulates expression of *DUR31*. (A) The intron structure of *DUR31* in *C*. *parapsilosis* is shown above the gel. Exons are depicted in green, introns in blue, and the TPP riboswitch in magenta. Splice sites are indicated with GU and AG. Stop codons in-frame with Exon 1 are represented by asterisks. Splice isoforms are illustrated using dotted gray lines. Spliced *DUR31* transcripts were identified by RT-PCR from cells grown in SC in the absence of thiamin (-Thi) or following the addition of 1 μM or 30 μM thiamin using primers CP_TPP_F2 and CP_TPP_R3. S = fully spliced, PS = partially spliced (at second GU) and U = unspliced products. S, PS and U products were confirmed by sequencing. We were unable to unambiguously determine the sequence of the band with slightly lower molecular weight than the U product in *C*. *parapsilosis DUR31*, and because only the two predicted spliced and one unspliced products are identified in RNA-seq data [19], we assume that it is an artifact. (B) The intron structure of *DUR31* in *Ogataea* (*Hansenula*) *polymorpha* is shown above the gel, with the same color scheme as in (A). Spliced *DUR31* transcripts were identified by RT-PCR from cells grown in SC in the absence of thiamin (-Thi) or following the addition of 30 μM thiamin using primers HpDUR31f2/HpDUR31r1.

While characterizing the small RNAs transcribed in the pathogenic yeast *Candida parapsilosis* and its relatives [19] we noted that the RNA structure predictor software Infernal [20] identified a potential TPP riboswitch in an intron of a poorly characterized gene *CPAR2_502100*, which we named *DUR31* after its ortholog in *Candida albicans* [21, 22] (Fig 1A). Prediction of a riboswitch was surprising, because until very recently, most reports suggest that riboswitches are absent from budding yeasts [10, 12, 23]. We therefore tested the effect of thiamin on splicing of *DUR31* in *C*. *parapsilosis*.

### Expression of *DUR31* is regulated by thiamin

The riboswitch in *DUR31* in *C*. *parapsilosis* is in an intron near the 5’ end of the gene (Fig 1A). The intron contains two potential 5’ donor splice sites, followed by the riboswitch, and a single 3’ acceptor site. The first 5’ donor site matches the *C*. *parapsilosis* 5’ donor consensus sequence (GTATGT), whereas the second has a non-consensus sequence (GTTGGA). In the absence of thiamin, most of the RNA is spliced at the first site, generating an mRNA (S) that encodes a full-length protein of 525 amino acids. In the presence of thiamin, the amount of unspliced mRNA (U) is increased. Some of the RNA is spliced at the second donor site (PS) in both the presence and absence of thiamin. The unspliced and partially spliced products do not encode a full-length protein, because of a premature stop codon after 58 amino acids, between the two 5’ splice sites.

**Fig 2:**
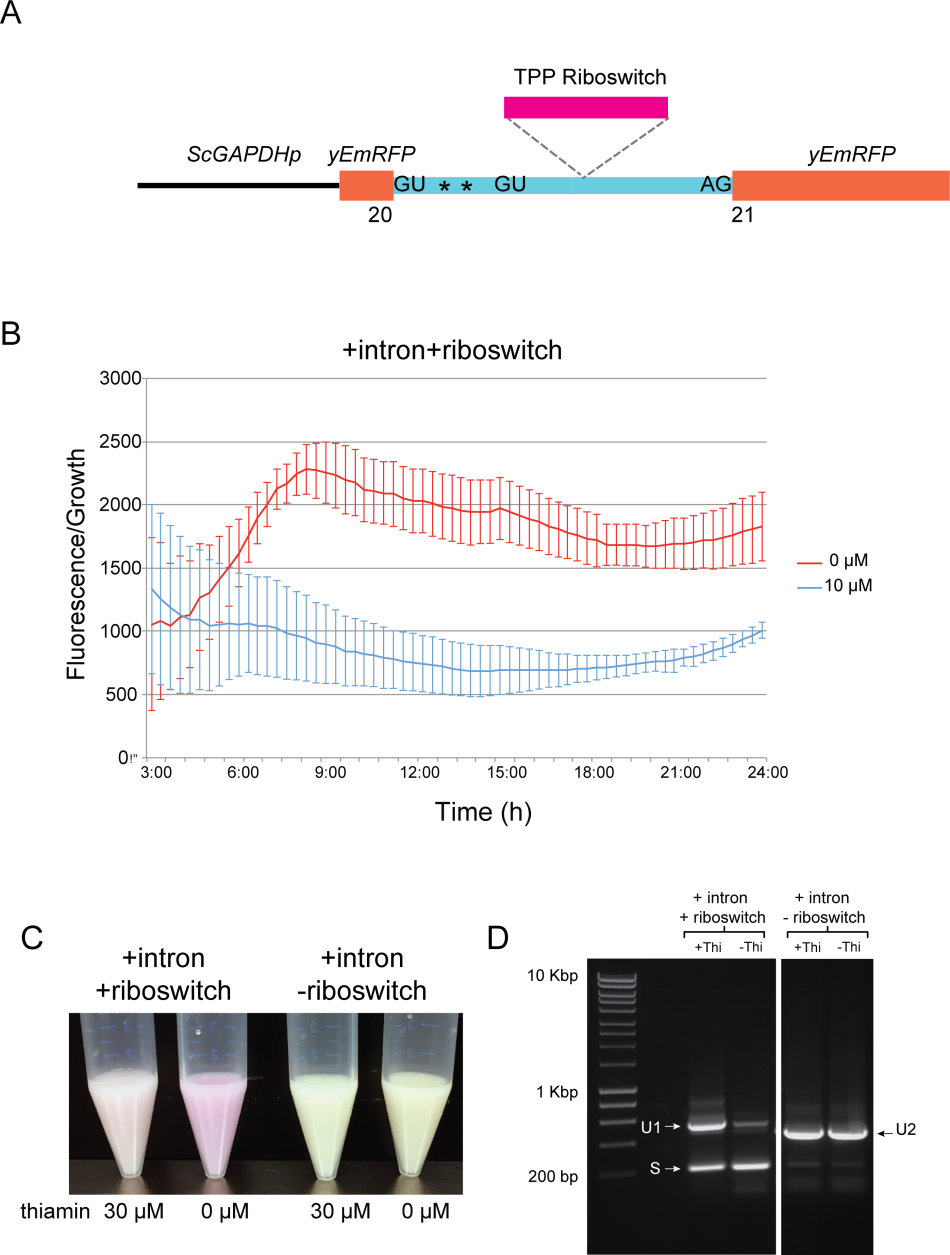
Thiamin regulates splicing of the *DUR31* intron from *C*. *parapsilosis*. (A) The intron from *C*. *parapsilosis DUR31* was synthesized with and without the riboswitch sequence and inserted after codon 20 in the purple fluorescent protein, yEmRFP, in a replicating plasmid. Expression was driven from the *S*. *cerevisiae* promoter *GAPDH*. Plasmids were transformed into *S*. *cerevisiae*. (B) Fluorescence levels and growth of *S*. *cerevisiae* BY4741 transformed with plasmids containing yEmRFP interrupted by the intron and riboswitch (+intron +riboswitch) was measured. Cells were grown in media with no thiamin or with 10 μM thiamin over 24 h. The y-axis represents the ratio of fluorescence to growth (A_600_), and x-axis displays time (h). Error bars show the error of propagation calculated from the standard deviation and mean from three replicate cultures. A_600_ measurements before 3 h are very low and highly variable. (C) Colors of cells spun down after 24 h growth in media in the absence or presence of thiamin. (D) Thiamin-dependent splicing requires the presence of the riboswitch. RNA was isolated from cells transformed with RFP interrupted by the intron and riboswitch (+intron +riboswitch) or with RFP interrupted by the intron with no riboswitch (+intron -riboswitch) and grown in the presence or absence of 30 μM thiamin for 5 h. Spliced (S) and unspliced (U1 and U2) products were identified by RT-PCR using primers RFPCheck_F and GapRFP_R. U2 is smaller than U1 because the intron without a riboswitch is shorter (by 99 bp) than the intron containing the riboswitch.

The difference in the total amount of spliced and unspliced mRNA in the presence and absence of thiamin could result from regulation of expression of *DUR31*, or from regulation of splicing by the riboswitch. To explore these possibilities, we introduced the intron from *C*. *parapsilosis* into the coding sequence of a purple fluorescent protein (yEmRFP) gene [24], so that it interrupts the ORF (+intron +riboswitch). This modified RFP was constitutively expressed from a *GAPDH* promoter on a plasmid in *S*. *cerevisiae*, thus removing any effect of thiamin on the endogenous *DUR31* promoter (Fig 2A). When cells containing the intron plus riboswitch construct are grown in the presence of thiamin, fluorescence levels remain at a low level. In cells transferred to medium without thiamin, fluorescence levels increase with respect to growth (Fig 2B). Pink/purple color is clearly higher in transformed *S*. *cerevisiae* cells grown overnight in the absence of thiamin, compared to cells grown in the presence of thiamin (Fig 2C).

In the presence of thiamin, most of the yEmRFP product from the intact intron is unspliced after 5 h (U1, Fig 2D), though some splicing does occur at the first donor site (S). There is no evidence of splicing at the second donor site, which is seen when the intron is in its native position in *C*. *parapsilosis* (Fig 1A). In the absence of thiamin, the amount of unspliced (U1) mRNA is greatly reduced (Fig 2D). The ratio of spliced/unspliced product is substantially different in the absence and presence of thiamin, suggesting that thiamin is regulating splicing, or regulating the stability of the unspliced product. The increase in fluorescence observed in Fig 2B in the absence of thiamin suggests that splicing, rather than stability, is regulated.

The experiment also shows that the *C*. *parapsilosis* TPP riboswitch is fully functional in *S*. *cerevisiae*, even though there are no riboswitches in this species. These results suggest that all the regulatory components are present in the intron.

### The riboswitch is required for splicing of the *DUR31* intron

We next made a version of the intron that maintains the donor and acceptor splice sites but does not include the riboswitch, and introduced it into yEmRFP at the same position (+intron -riboswitch construct, (Fig 2)). We predicted that this construct would be spliced both in the presence and absence of thiamin. However, in *S*. *cerevisiae* cells transformed with the construct, fluorescence levels approached zero, and cells are completely white (Fig 2C). We used RT-PCR to show that the intron lacking a riboswitch (+intron–riboswitch) is not spliced from yEmRFP, even when there is no thiamin present (U2, Fig 2D). When the riboswitch is present (+intron +riboswitch) it acts as an “off” switch (increased unspliced RNA and reduced expression in the presence of thiamin/TPP). Our results suggest that the riboswitch is also required for splicing to occur. It is possible that shortening the intron changed the accessibility of the donor and/or acceptor splice sites. However, all intron-associated features are still present in the two constructs, including the probable branch site (TACTAAC).

### Dur31 proteins are thiamin transporters

**Fig 3:**
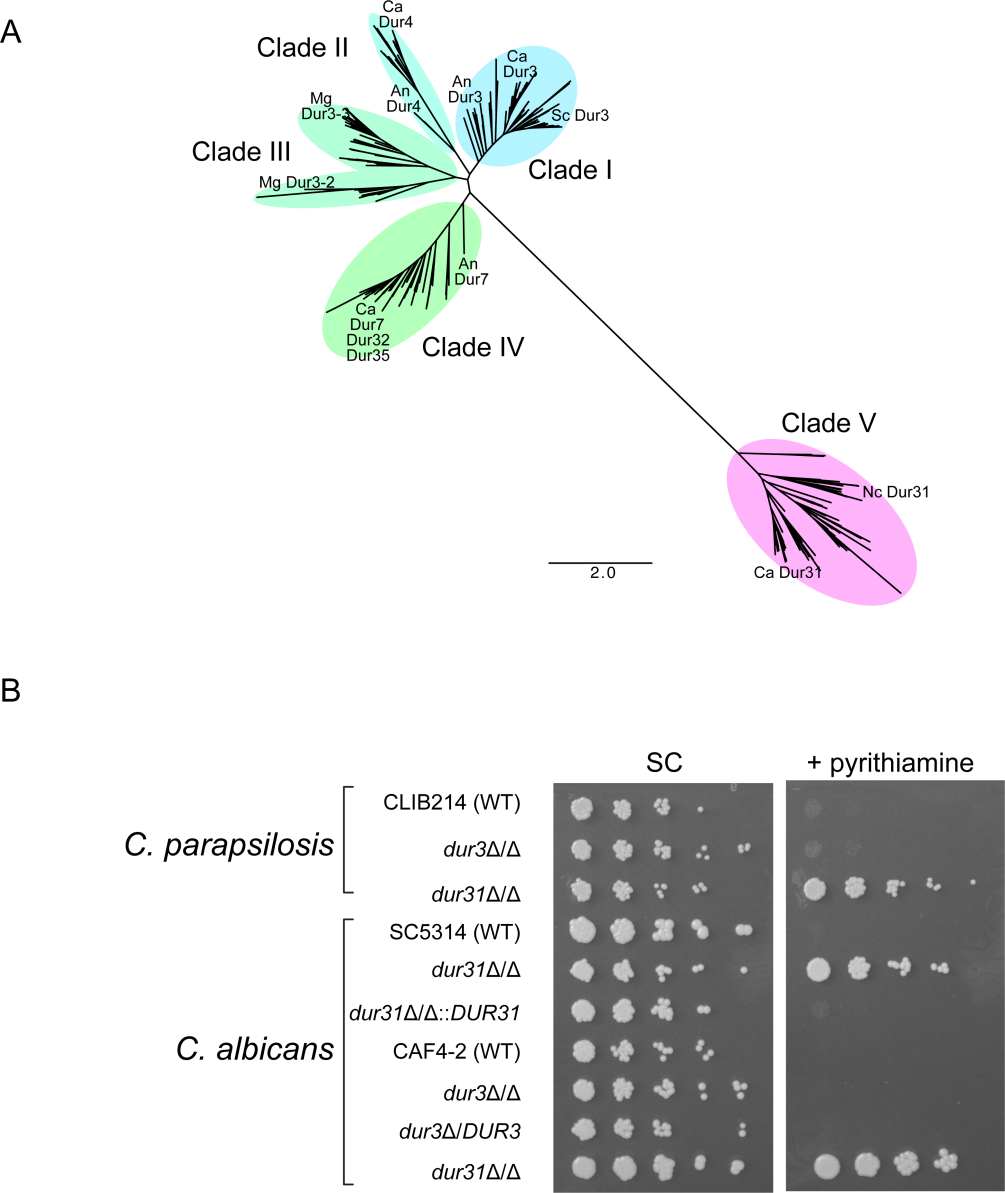
Dur31 proteins are thiamin transporters. (A) Dur31 and Dur3 families in Ascomycota fungi. Paralogous Dur3 families are shown in Clades (I-IV) and Dur31 paralogs in Clade V. A full version of the tree showing all species names is provided in supplementary S5 Fig. An–*A*. *nidulans*, Ca–*C*. *albicans*, Mg–*M*. *guilliermondii*, Nc–*N*. *crassa*, Sc–*S*. *cerevisiae*. An–*A*. *nidulans*, Ca–*C*. *albicans*, Mg–*M*. *guilliermondii*, Nc–*N*. *crassa*, Sc–*S*. *cerevisiae*. Sequences were aligned using Muscle [48] and the tree was constructed using RAxML [49]. (B) Dur31 is a thiamin transporter. *C*. *albicans* and *C*. *parapsilosis* CLIB214 (WT), *dur3*, *and dur31* deletion strains were spotted in increasing dilutions on Synthetic Complete (SC) medium, and SC medium supplemented with pyrithiamine hydrobromide (10 μM). Deleting *DUR31* allows growth in the presence of the toxin pyrithiamine in both species, and restoring *DUR31* in *C*. *albicans* complements the resistance phenotype. Derivatives of *C*. *albicans* SC5314 are from Mayer et al. [22] and *C*. *albicans* CAF4-2 are from Kumar et al.[21]. The *C*. *parapsilosis* deletion strains were made as part of this study.

Because splicing of the *DUR31* intron is regulated by thiamin, we predicted that the protein is likely to be involved in thiamin biosynthesis or transport. Dur31 is a homolog of *N*. *crassa NCU01977*, in which splicing is also regulated by a TPP riboswitch [11]. Dur31/NCU01977 belong to the solute carrier 5 transporter family. These proteins contain SSF domains, which indicate that they act as sodium-solute symporters [25]. Dur31 is related to Dur3 in *S*. *cerevisiae* (average identity is 12.6% in *C. parapsilosis*), and Dur3 homologs in both *S*. *cerevisiae* and *C*. *albicans* transport urea and polyamines [21, 22, 26, 27]. In order to determine the relationship between Dur31 and the large family of related proteins in the Ascomycota, we searched for homologs using a Hidden Markov Model (HMM) generated using Dur31 from 14 Saccharomycotina species. The model identified both Dur3 and Dur31 homologs. By phylogenetic analysis (Fig 3A), we identified several clades, including at least four that are relatively closely related to Dur3 (Fig 3A). Clade I includes proteins from *S*. *cerevisiae* and *C*. *albicans* that are known to transport spermidine and other polyamines, such as Dur3 itself [21]. Clade II contains homologs of Dur4, an uncharacterized protein in *C*. *albicans* that is assumed to be a urea transporter [28]. Clade III, which may contain two sub-clades, contains uncharacterized proteins which we have named here as Dur3-2 and Dur3-3. Clade IV contains homologs of Dur7, Dur32, and Dur35 from *C*. *albicans*, again assumed to be urea transporters [29]. In contrast to clades I-IV, clade V is only distantly related to the others. Clade V contains all Dur31 orthologs, including *C*. *parapsilosis* Dur31 and *N*. *crassa* NCU01977 (NcDur31).

The gene duplication that formed clade V is old, and predates the divergence between the Saccharomycotina and the Pezizomycotina lineages (Fig 3A). For example, there are *N*. *crassa* (Pezizomycotina) genes in clades I and V, and *A*. *nidulans ge*nes in clades, I, II and IV (Fig 3A). Mukherjee et al [12] identified orthologs of *DUR31/NCU01977* in basidiomycetes and in oomycetes, some of which contain TPP riboswitches, suggesting that it is an ancient gene. However, Dur31 is completely absent from *S*. *cerevisiae* and its close relatives (see below).

To characterize the roles of *DUR3* and *DUR31*, we deleted the genes in *C*. *parapsilosis*, and we acquired equivalent deletion strains of *C*. *albicans* [21, 22]. Deleting *DUR31*, but not *DUR3*, allows growth of both *C*. *parapsilosis* and *C*. *albicans* in the presence of pyrithiamine, a toxic analog of thiamin (Fig 3B) [30]. Only *C*. *parapsilosis* and not *C*. *albicans DUR31* contains a riboswitch. The lack of toxicity is therefore not due to an interaction of pyrithiamine pyrophosphate (PTPP) with the riboswitch. The simplest interpretation is that deleting *DUR31* prevents uptake of both thiamin and pyrithiamine, allowing growth in the presence of the toxic compound.

### Distribution of thiamin transporters in the Saccharomycotina

Active thiamin transport has been extensively studied in *S*. *cerevisiae*, where the transporter is Thi7 [31]. *THI7* has undergone a specific gene amplification in *S*. *cerevisiae*, resulting in three members; *THI7* (also called *THI10*), *THI72*, and *NRT1* [31, 32]. In *S*. *cerevisiae* Thi7 is a high affinity transporter of thiamin, and Thi72 and Nrt1 are low affinity transporters. *THI72* and *NRT1* are not present in other yeast species.

**Fig 4:**
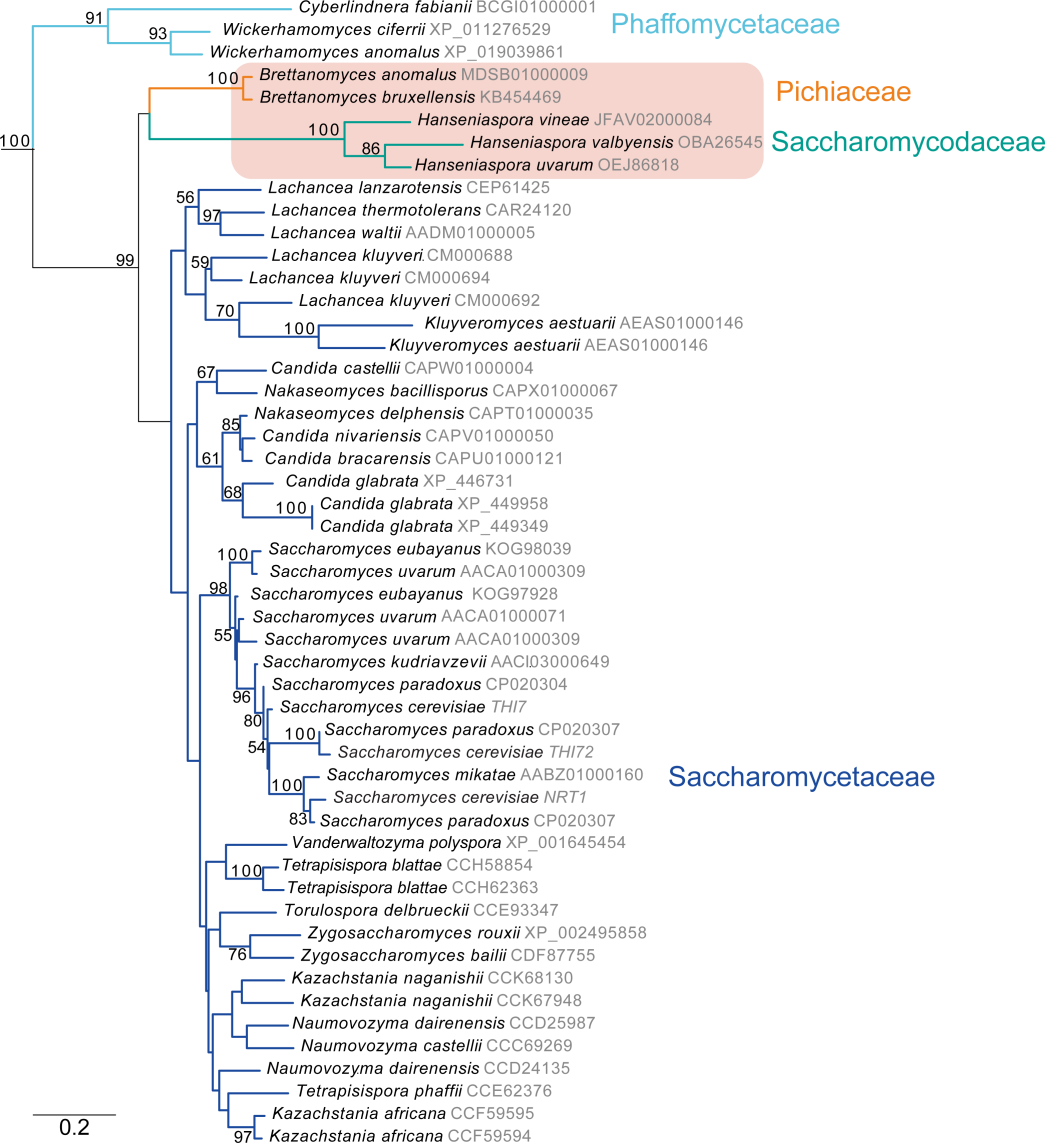
Thi7 orthologs in the Saccharomycotina. Thi7 orthologs were identified as shown in S1 Fig, and the tree is rooted using paralogous genes. The amino acid sequences of predicted Thi7 homologs were aligned using Muscle (v3.8.31, [48]), and a phylogenetic tree was constructed using RAxML [49]. Branch support is indicated using bootstrap values (from 100. Only values >50 are shown). Protein accession numbers are shown, or where unavailable, contig names are shown. A possible HGT event in *Brettanomyces* is highlighted in a box. The species are colored using the format in Fig 5 (Saccharomycetaceae (dark blue), Saccharomycodaceae (sea green), Phaffomycetaceae (cyan), Yarrowia (red), Debaryomycetaceae/Metschnikowiaceae (yellow), Pichiaceae (orange).

Thi7 belongs to the Major Facilitator Superfamily (MFS), which is structurally unrelated to the SSF domain family represented by Dur31. Specifically, Thi7 belongs to a subset of MFS transporters that includes Dal4 (allantoin permease), Fur4 (uracil permease), and Fui1 (uridine permease). Because MFS is a large family, we first used phylogenetic analysis to separate the Thi7 orthologs from related proteins (S1 Fig). We then constructed a tree of these Thi7 orthologs (Fig 4). Thi7 is entirely absent from the Pezizomycotina. In the Saccharomycotina, there are Thi7 orthologs in species within the Saccharomycetaceae, the Saccharomycodaceae, and the Phaffomycetaceae, but not in other lineages such as *Yarrowia* and the Debaryomycetaceae /Metschnikowiaceae clades (Fig 4). Two *THI7* genes were also identified in the Pichiaceae species, *Brettanomyces bruxellensis* and *Brettanomyces anomalus* (Fig 4). The *Brettanomyces* orthologs are more closely related to Thi7 proteins from the *Hanseniaspora* (Saccharomycodaceae) species.

### Distribution of riboswitches

**Fig 5:**
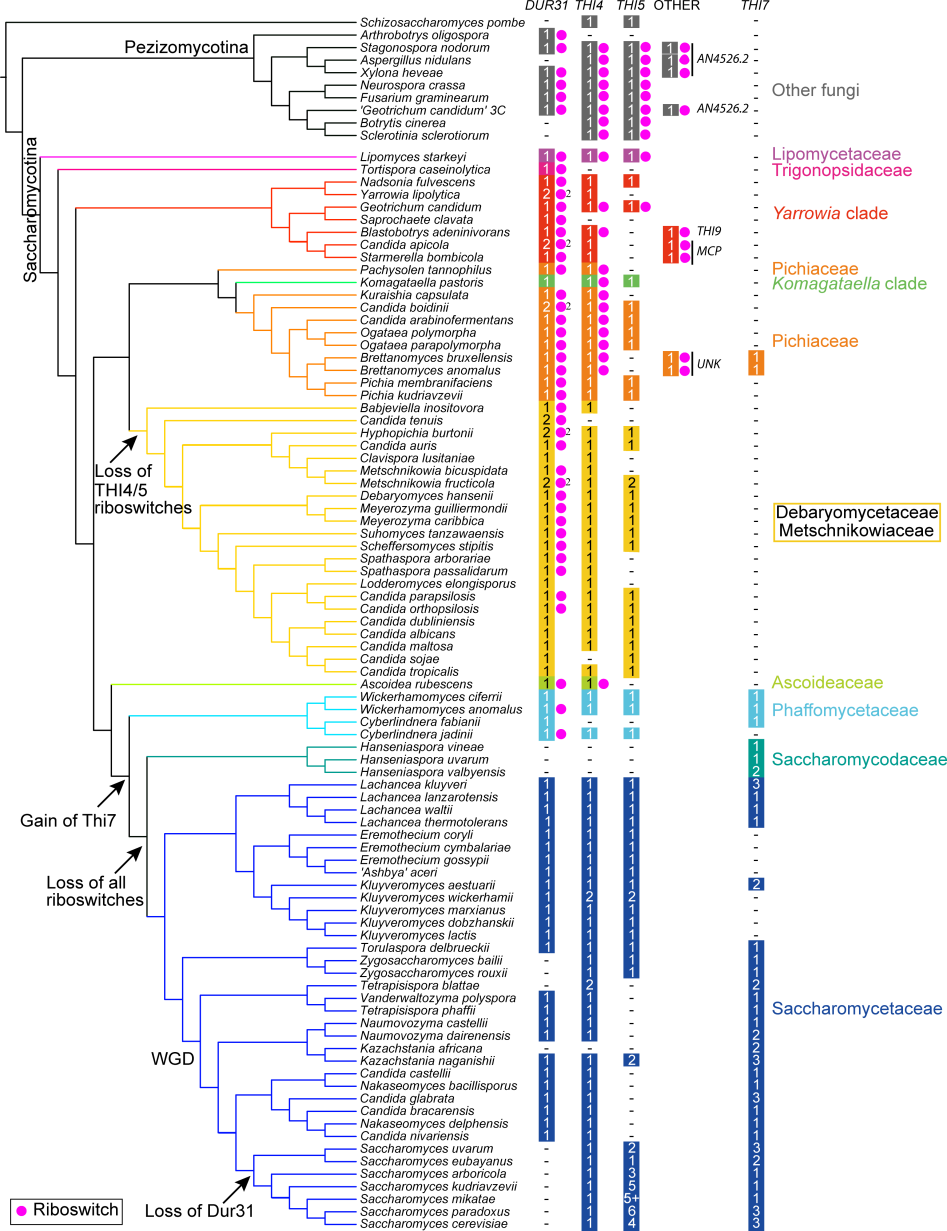
Identification of riboswitches and thiamin metabolism and transport genes in the Saccharomycotina. The presence of a riboswitch is indicated with a pink dot, and the number of orthologs of thiamin biosynthesis (*THI4* and *THI5*) and transport (*DUR31* and *THI7*) genes in each species is shown. The number of *THI5* orthologs in *Wickerhamomyces anomalus* is not clear, because this species is a diploid hybrid. In nine species there are riboswitches in genes that are not orthologs of *DUR31*, *THI4*, *THI5*, or *THI7*. These are indicated under “other”, and are labeled as *THI9*, *AN4526*.*2*, *MCP* or unknown. The presence of orthologs of these genes without riboswitches is not recorded for other species. Absence of *DUR31*, *THI4*, *THI5*, or *THI7* orthologs is shown with a dash. The most likely timings of gene or riboswitch loss are shown in the branches of the tree. The phylogeny and clade names of 86 Saccharomycotina and 10 outgroup species is taken from Shen et al [34]. WGD = the whole genome duplication.

Riboswitches were generally assumed to be missing from budding yeasts (Saccharomycotina) [23], although a very recent study has identified some in a small number of species [12]. To examine the distribution of riboswitches in yeasts, we examined 86 genomes of species from the Saccharomycotina and 10 outgroup species [33, 34]. We used the software Infernal to predict riboswitches, together with a detailed manual analysis of associated open reading frames (Fig 5, S1 Data Set, see Methods). As expected, many of the riboswitches we found are in genes known to be involved in thiamin metabolism.

Riboswitches were most commonly found in *DUR31* homologs (S2 Fig). TPP riboswitches are present in introns of most of the *DUR31* homologs in species outside the family Saccharomycetaceae, including in many species in the Debaryomycetaceae/Metschnikowiaceae (CUG-Ser) clade, the Pichiaceae, and the *Yarrowia* clade (Fig 5). Riboswitches are also present in *DUR31* in many *Candida* species (as well as *Candida parapsilosis*), but are missing from the well-studied *Candida albicans* (Fig 5). Some of the other *DUR31* orthologs in this clade contain introns near the 5’ end of the gene, but have no riboswitch (e.g. *Lodderomyces elongisporus*).

Not all of the *DUR31* orthologs in the Saccharomycotina have obvious alternative donor splice sites like *CPAR2_502100*. However, where a riboswitch is present, splicing, or expression, is probably controlled by thiamin. We characterized expression of *DUR31* from *Ogataea polymorpha* (a Pichiaceae species), where only one donor and one acceptor site was identified (Fig 1B). A fully spliced product is present only in the absence of thiamin, and only the spliced product encodes a full-length protein (Fig 1B). Thiamin therefore represses the production of a functional Dur31 protein in both *C*. *parapsilosis* and *O*. *polymorpha* by repressing production of a functional spliced isoform. Some regulation may be exerted at the level of transcriptional regulation, similar to the repression of thiamin synthesis genes in *S*. *cerevisiae* [35].

TPP riboswitches were rarely found in thiamin biosynthesis genes (*THI4*, *THI5*) in budding yeast species, unlike in Pezizomycotina [10–12, 23]. Only two were identified in *THI5*, both in basal lineages (*Geotrichum candidum* and *Lipomyces starkeyi*) (Fig 5). There are riboswitches in introns in *THI4* in family Pichiaceae, some of the *Yarrowia* clade, and *Ascoidea rubescens*. However, riboswitches appear to have been lost from thiamin biosynthesis genes in the Debaryomycetaceae /Metschnikowiaceae.

TPP riboswitches were also identified in a small number of genes that encode neither known thiamin biosynthesis enzymes nor Dur31 homologs (Fig 5). These include genes in *Stagonospora nodorum*, *Xylona heveae*, *Aspergillus nidulans* (AN4526.2), and a filamentous fungus (incorrectly identified as *Geotrichum candidum* 3C [36]), that are predicted to encode nucleoside transporters, and share some similarities with *S*. *cerevisiae* Tpn1, a transporter of pyridoxine (Vitamin B6), and Fcy21, a member of the purine-cytosine permease family whose function is unknown [37]. More fungal homologs belonging to this class were identified by Moldovan et al [23] and were categorized as “putative transporters”. They are predicted to play some role in thiamin metabolism. One riboswitch-containing gene in *Blastobotrys (Arxula) adeninivorans* is a homolog of Thi9, the thiamin transporter in *Schizosaccharomyces pombe* [17]. TPP riboswitches are also present in other genes in *Candida apicola* and *Starmerella bombicola* that are related to the monocarboxylate porter (MCP) family, part of the MFS superfamily. Finally, riboswitches were predicted in small transcripts with little obvious protein coding potential in *Brettanomyces anomalus* and *Brettanomyces bruxellensis* (S3 Fig).

No TPP riboswitches were predicted in any genes in Saccharomycetaceae species (Fig 5).

## Discussion

We found that riboswitches are common in orthologs of *DUR31*/ *NCU01977* in budding yeasts (Fig 5) and we showed that Dur31 transports thiamin in *C*. *parapsilosis* and *C*. *albicans*, a function that is likely conserved among many fungal species. Dur31 is an ancient gene, that predates the separation of the Pezizomycotina and the Saccharomycotina (Fig 5), and was probably present in the ancestor of fungi and oomycetes [12]. It has been lost from *Schizosaccharomyces pombe*, a member of the Taphromycotina, which lies at the base of the Saccharomycotina (Fig 5). In *S*. *pombe*, thiamin is transported by Thi9, which is more closely related to amino acid transporters [38]. Thi9 orthologs in other Taphrinomycotina species, and in Pezizomycotina (filamentous fungi) and more distantly related Basidiomycetes, also contain riboswitches [23]. *B*. *adeninivorans* retains both *DUR31* and *THI9*, and riboswitches are present in both (Fig 5).

Another thiamin transporter, Thi7 from the MFS family, is present in species within the Saccharomycetaceae, the Saccharomycodaceae and the Phaffomycetaceae, and in two Pichiaceae species (*B*. *bruxellensis* and *B*. *anomalus*) (Fig 5, Fig 4). Analysis of the phylogenetic relationship of the *THI7* homologs suggests that it may have arisen recently in the ancestor of the Phaffomycetaceae/Saccharomycodaceae/Saccharomycetaceae and its presence in Brettanomyces may result from Horizontal Gene Transfer (HGT), most likely from a Saccharomycodaceae species (Fig 4).

**Fig 6:**
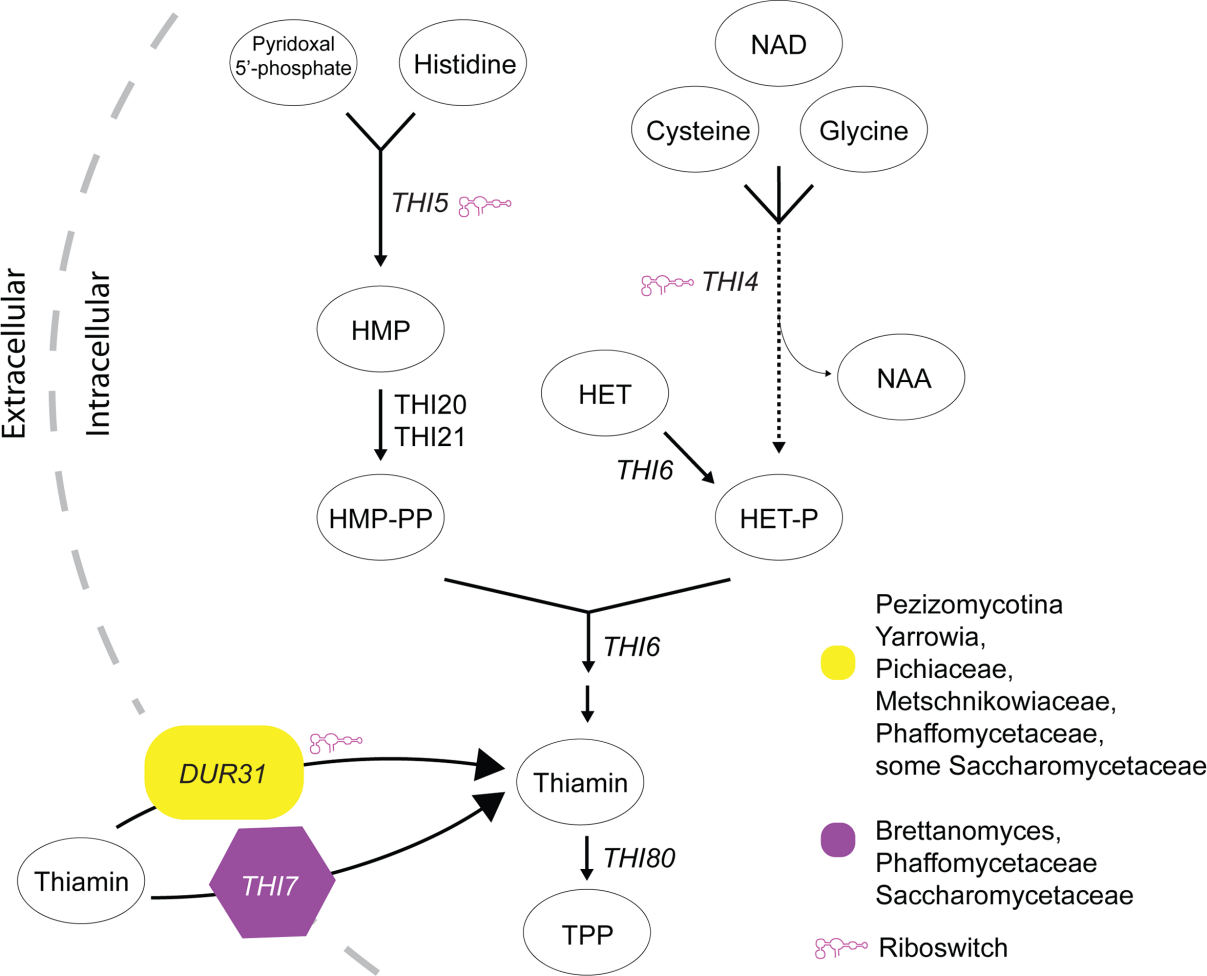
Thiamin biosynthesis and transport in the Ascomycota. Thiamin is either imported from outside the cell, or synthesized endogenously via both Thi4 and Thi5 pathways, and then converted into the biologically active form TPP. Thiamin can be transported by Dur31 and/or Thi7, and possibly by other transporters including Thi9 and members of the MCP family. The species distribution of Dur31 is indicated in yellow, and Thi7 in purple. The pink riboswitch symbol indicates genes that contain riboswitches in some species (see Fig 5).

Dur31 has been lost from *S*. *cerevisiae* and its close relatives, and independently from other lineages including the Saccharomycodaceae, and the *Zygosaccharomyces*/*Torulaspora* branch. In *S*. *cerevisiae* Thi7 is the main transporter of thiamin [32], and it is likely that Thi7 orthologs transport thiamin in the other species also. There have been some independent losses of Thi7 (for example in the *Eremothecium* lineage, and in *Cyberlindnera jadinii*). All of the budding yeast species that lack Thi7 contain Dur31 (Fig 5). We predict that Dur31 is the major thiamin transporter in these species (Fig 6). It is not known what selective pressure drove the displacement of Dur31 by Thi7. Many species retain both Dur31 and Thi7 (e.g. *Lachancea kluyveri*), but only one (*Wickerhamomyces anomalus*) has both a riboswitch-containing intron in *DUR31*, and *THI7*. The shift from *DUR31* to *THI7* may therefore coincide with a move from riboswitch-mediated thiamin-dependent expression to thiamin regulation at the promoter [32] (Fig 5, Fig 6).

The exact mechanism of action of the thiamin riboswitch in *C*. *parapsilosis* Dur31 is currently unknown. All of the required regulatory sequences are contained within the intron, because thiamin-dependent splicing occurs when this region is introduced into a yEmRFP coding sequence in *S*. *cerevisiae* (Fig 2). The *C*. *parapsilosis* intron is 351 bp, whereas the intron in *N*. *crassa* is 602 bp; it is therefore unlikely that the same long-range interactions proposed for *N*. *crassa NCU01977* occur in *C*. *parapsilosis DUR31* [11]. However, the *DUR31* riboswitch appears to be required for splicing, because when it is removed from the intron splicing does not occur. Mukherjee et al [12] suggest that in introns like this (which they call Type III), access of the splicing machinery to the first and second donor sites is regulated by the riboswitch, without involving a long range interaction. We see little evidence that access to the second donor site is regulated by thiamin in *C*. *parapsilosis DUR31* (Fig 1A), and some genes have only one obvious donor site (Fig 1B). However, unspliced products contain stop codons (Fig 1), and so are likely to be subject to nonsense-mediated decay [39]. The riboswitch may therefore control access to the first donor site.

Moldovan et al [23] identified 5 groups of fungal genes that contain riboswitches (although they failed to identify riboswitches in Saccharomycotina species). We identified the same 5 genes–*THI4*, *THI5*, *DUR31* (*NCU01977*), *THI9*, and a putative transporter family including *A*. *nidulans* AN4526.2. We also identified a sixth group, represented by *MCP* in *S*. *bombicola* and *C*. *apicola*. Four of the ortholog groups have transporter domains (*DUR31*, *THI9*, *AN4526*.*2* and *MCP*), and the first two include members that have now been shown to transport thiamin. It is therefore likely that the second two also transport thiamin or thiamin metabolites. The AN4526.2 family may be restricted to species outside the Saccharomycotina, and the distribution of the MCP family is unknown. The function of the additional riboswitches in *Brettanomyces* species is not clear. They are not adjacent to any obvious open reading frame, though they do lie within 1.5 kb of a putative thiamin biosynthesis gene.

In the Saccharomycotina, riboswitches are rarely found in genes encoding thiamin biosynthesis enzymes; they are present in *THI5* in only two species, and they are completely absent from *THI4* in the Debaryomycetaceae/Metschnikowiaceae. In addition, all TPP riboswitches have been lost from the sequenced isolates in the family Saccharomycetaceae, including *S*. *cerevisiae*. The loss of riboswitches in the thiamin synthesis genes may be associated with a switch to thiamin-dependent regulation at the level of transcriptional initiation. In *S*. *cerevisiae*, expression of thiamin synthesis genes is strongly regulated in response to thiamin levels, and requires the activity of the transcription factors *THI2*, *THI3* and *PDC2* [35, 40]. *THI2* and *THI3* are not conserved outside the Saccharomycetaceae, and the role of *PDC2* orthologs in regulating thiamin-dependent expression has not been investigated in species outside this clade. The relative contribution of riboswitches versus transcription factor activity in *Candida* species therefore remains to be determined.

Our analysis allowed us to identify loss of thiamin biosynthesis genes, as well as gain and loss of transporters and riboswitches. The alternative routes that yeast use to obtain thiamin are shown in Fig 6. The biosynthesis of thiamin is well studied in *S*. *cerevisiae*, and involves the convergence of two separate pathways; synthesis of HET-P, which requires Thi4, and synthesis of HMP-PP, which requires Thi5 (Fig 6). *THI4* and *THI5* are present in most fungi, but several species have lost both genes, including three *Hanseniaspora* species, and *Kazachstania africana* (Fig 5). These species probably cannot synthesize thiamin or thiamin precursors. This idea is supported by reports that *Hanseniaspora valbyensis* cannot synthesize the B vitamins biotin and pantothenate, and can only partially synthesize thiamin, niacin and pyridoxine [41]. Other *Hanseniaspora* and *Kazachstania* species also require added B vitamins, including thiamin, for growth [42, 43]. An additional 27 species have lost either *THI4* or *THI5* (Fig 5). Some that are missing only *THI5* (e.g. *Candida glabrata*, [44]) exhibit thiamin auxotrophy that can be complemented by supplementing with HMP [45]. These species most likely import HMP using the same transport mechanisms as thiamin (using Thi7 or Dur31). Loss of *THI4* is rarer, and the only species that has lost *THI4* but not *THI5* is *Candida sojae*. It is not clear if this reflects a true gene loss, or an error in the genome assembly.

In summary, we find that Dur31 is one of several types of thiamin transporters, that has been present since the divergence of oomycetes and fungi [12]. There appears to be a high turnover of thiamin transporters in fungi. There has also been a gradual loss of riboswitches in yeasts, where they are most common in *DUR31*. It is likely that riboswitches also regulate expression of other thiamin transporters (such as Thi9, and an additional putative transporter that may be restricted to species outside the Saccharomycotina), but these remain to be experimentally characterized.

## Methods

All strains used are listed in S1 Table, all primers in S2 Table. Identified genes and riboswitches are listed in S1 Data Set.

Data availability: There is no restriction on availability of constructs or data described.

Code availability: All custom scripts are available at https://github.com/GiantSpaceRobot/Riboswitch.

### Strains, media and growth

All strains are listed in S1 Table. *S*. *cerevisiae* BY4741, *C*. *albicans*, *C*. *parapsilosis*, and *O*. *polymorpha*, were maintained on YPD (2% glucose, 2% peptone, 1% yeast extract) and *E*. *coli* DH5α on LB (1% tryptone, 1% NaCl, 0.5% yeast extract) or LB supplemented with 100 μg/mL ampicillin. SC-uracil agar (2% glucose, 2% Bacto agar, 0.5% ammonium sulfate, 0.19% YNB without amino acids or ammonium sulfate (Formedium), 0.1926% Synthetic Complete -Uracil dropout mix (Formedium)) was used to select for transformants. To investigate alternative splicing, strains were grown in SD-thiamin (2% glucose, 0.5% ammonium sulfate, 0.171% YNB minus thiamin (Sunrise Science Products)) supplemented with 1 or 30 μM thiamin where indicated. Media for *S*. *cerevisiae* BY4741 was also supplemented with leucine (380 mg/L), methionine (76 mg/L), and histidine (76 mg/L). Pyrithiamine hydrobromide was added at a final concentration of 10 μM, and agar at 2% where indicated.

### Growth and fluorescence of *S*. *cerevisiae* BY4741

200 μL SD-Thiamin and 200 μL SD-Thiamin supplemented with 10 μM thiamin were added to triplicate wells in a round-bottom 96 well plate. 10 μL cells (A_600_ of 2) was added to each and the plate was covered with a Breathe-Easy gas-permeable membrane (Sigma-Aldrich). This was placed in a Synergy plate reader and maintained at 30°C. The A_600_ and fluorescence were measured at time zero, then every 20 min for 24 h with vigorous shaking. For fluorescence, excitation was measured using 590/20 nm filters, where 590 nm denotes the arithmetic mean of the wavelength at 50% of peak transmission, and 20 nm denotes the full-width at half the maximum (FWHM) transmission, which is the bandwidth at 50% of peak transmission. Emission was measured with 645/40 nm filters. The mean of the media-only wells was subtracted from each replicate. The error bars show the standard deviation calculated from the error of propagation (s(x/y) = (x/y)(sqrt(sumsq(s(i)/m(i))))).

### Identification of *DUR31*, *THI4*, *THI5* and *THI7* homologs from Saccharomycotina

PFAM hidden Markov models (HMMs) for Thi4 and Thi5/Nmt1 were obtained from the Pfam database [46]. HMMs for Dur31 or Thi7 were constructed using HMMER [47] from the relevant orthologs from a number of the species in Fig 5 (Dur31 proteins: *C*. *parapsilosis*, *C*. *orthopsilosis*, *L*. *elongisporus*, *D*. *hansenii*, *M*. *guilliermondii*, *S*. *passalidarum*, *S*. *stipitis*, *C*. *tenuis*, *C*. *albicans* SC5314, *C*. *albicans* WO-1, *C*. *dubliniensis*, *C*. *tropicalis*, *C*. *lusitaniae*, *O*. *polymorpha*, *O*. *parapolymorpha*. Thi7: *V*. *polyspora*, *N*. *dairenensis* (2), *N*. *castellii*, *K*. *naganishii* (2), *K*. *africana*, *C*. *glabrata*, *S*. *cerevisiae*, *Z*. *rouxii*, *T*. *delbrueckii*, *L*. *kluyveri*, *L*. *thermotolerans*, *L*. *waltii*, *T*. *blattae*, *T*. *phaffii*). These were aligned with Muscle (v3.8.31) [48]. HMMER was used to screen the proteomes of all species in Fig 5, followed by manual inspection of phylogenetic gene trees created by RAxML [49]. ORFs were predicted using the Pico_Galaxy tool get_orfs_or_cdss.py where no proteome was available. Gene locations are listed in S1 Data Set.

### Identification of riboswitches

Genomes of 96 species were obtained from GenBank, NCBI Genome, the Candida Gene Order Browser [50], and the Joint Genomes Institute. Riboswitches were identified using cmsearch in Infernal with the RFAM TPP Riboswitch covariance model (RF00059) [20]. In species where riboswitches were predicted in regions that were not adjacent to *DUR31*, *THI4*, and *THI5* orthologs (*S*. *nodorum*, *A*. *nidulans*, *X*. *heveae*, *G*. *candidum* 3C, *B*. *adeninivorans*, *C*. *apicola*, *S*. *bombicola*, *B*. *bruxellensis*, and *B*. *anomalus*), the associated genes (e.g. *AN4526*.*2*, *THI9*) were identified by examination of the surrounding sequences. Only two of the three previously predicted *N*. *crassa* riboswitches [10, 11] were above the threshold predicted by Infernal. We identified the third riboswitch by selecting putative riboswitch predictions that were adjacent to HMMER predictions for thiamin biosynthesis genes. Applying the same strategy to all genomes did not identify any additional riboswitches near *DUR31*, *THI4*, *THI5*, *THI9* or *AN4526*.*2* homologs in any other species. The full list of riboswitches is provided in S1 Data Set.

### Deletion of *C*. *parapsilosis DUR3* and *DUR31*

*DUR3* (*CPAR2_105530*) and *DUR31* (*CPAR2_502100*) were deleted in *C*. *parapsilosis* CPL2H1 (*leu2−/his1−*) by replacing one allele of each with *HIS1* from *C*. *dubliniensis* and the second with *LEU2* from *C*. *maltosa* as described in Holland et al. [51]. Approximately 500 bases were amplified upstream and downstream of *DUR3* using primers CpDUR3_1/ CpDUR3_3, and CpDUR3_4/CpDUR3_6, and from *DUR31* using primers CpDUR31_1/ CpDUR31_3, and CpDUR31_4/CpDUR31_6 and joined to *CdHIS1* and *CmLEU2* by fusion PCR (S2 Table).

### Plasmid construction

The backbone of the pRS316-GAP-yEmRFP plasmid [24] was amplified using primers RFP_Lin-1_F and RFP_Lin-1_R (S2 Table). This plasmid contains a *URA3* selectable marker, 2-micron origin of replication, and yEmRFP. The intron from *C*. *parapsilosis DUR31* and a version without the riboswitch were synthesized using Gblocks (Integrated DNA Technologies, S4 Fig). These were amplified using primers Cpi-1_F and Cpi-1_R (S2 Table), which contain sequences that overlap with RFP_Lin-1_F and RFP_Lin-1_R. The native intron, and the intron without a riboswitch were joined to linearized pRS316-GAP-yEmRFP by gap repair in *S*. *cerevisiae* BY4741 [52], inserting the intron after amino acid 20 of RFP, and generating pPD-yRFPcpi and pPD-yRFPcpiNR. The plasmid were rescued from *S*. *cerevisiae* by transforming into *E*. *coli* and the sequence of the intron and surrounding regions was confirmed using Sanger sequencing.

### RT-PCR

Cells were first grown in YPD (or SC-uracil for *S*. *cerevisiae* BY4741) overnight, diluted to an A_600_ of 0.2 in 20 mL SD-Thiamin +/- additional thiamin, and for *C*. *parapsilosis* RNA was isolated after 5 h using an Isolate II Mini kit protocol (Bioline), following the manufacturers’ instructions except that 1 μg RNA in a total volume of 20 μL was treated with 1 μL DNase and 1 μL DNase buffer (Invitrogen) for 5 min at room temperature, followed by 1 μL DNase inactivation reagent and incubation at 65°C for 10 min. To generate cDNA, 4 μL of DNase-treated RNA and Oligo dT (Promega, final concentration of 20 μg/mL) was made up to 5 μL using water and incubated at 70°C for 10 min. 20 μL nuclease-free water with final concentrations of 1X MMLV-RT Buffer, 2 units/μL of RNasin, and 500 μM dNTPs were added to duplicate tubes. MMLV-RT was added to one set of duplicate tubes at a final concentration of 20 units/μL, and the same volume of water to the other set of duplicate tubes as a control to determine DNase-treatment success. The tubes were incubated at 37 C for 1 hr, followed by 2 min at 95 °C. RNA was extracted from *O*. *polymorpha* using hot-acid phenol, and SuperScript III Reverse Transcriptase (Invitrogen) was used for cDNA synthesis. Primers CP_TPP_F2/CP_TPP_R3 were used to characterize splicing in *C*. *parapsilosis*, HpDUR31f2/HpDUR31r1 in *O*. *polymorpha* (Fig 1), and RFPcheck_F/RFP_R in *S*. *cerevisiae* BY4741 + pPD-yRFPcpi (S2 Table).

## Supporting information

S1 Fig Phylogeny of Thi7 and related proteins from Saccharomycotina species. 16 Thi7 homologs from Saccharomycotina species were used to generate a Thi7 HMM, which was used with HMMER to search all species from Fig 5. The amino acid sequences of predicted Thi7 homologs were aligned using Muscle (v3.8.31), and a phylogenetic tree was constructed using RAxML. Thi7 is amplified in *S*. *cerevisiae* as shown (Thi7, Thi72, Nrt1). The three related *S*. *cerevisiae* proteins Fui1, Dal4, and Fur4 are also shown. The arrow shows the most-likely cut-off for Thi7 orthologs. The species are colored using the format in Fig 5 (Saccharomycetaceae (dark blue), Saccharomycodaceae (sea green), Phaffomycetaceae (cyan), Yarrowia (red), Debaryomycetaceae/Metschnikowiaceae (yellow), Pichiaceae (orange), Pezizomycotina (gray).
(PDF)

S2 Fig Alignment of all riboswitches predicted in *DUR31* genes from the species shown in Fig 1. The alignment was generated using T-Coffee [53] and visualized using SeaView [54]. The predicted riboswitch secondary structure is shown below, where ‘><‘ symbols indicate potential complementary nucleotides, ‘P1/P2’ shows the riboswitch stems, and ‘ …’ depicts loops and other unmatched nucleotides. Stem P3 is highly variable, as previously reported [7].
(PDF)

S3 Fig The TPP riboswitch in *Brettanomyces bruxellensis* is in a region with poor protein-coding potential. The gray, cyan, and magenta bars represent genomic DNA. The gray region depicts DNA outside the TPP riboswitch intron, the cyan depicts DNA inside the intron, and the magenta region depicts the TPP riboswitch. Potential splice isoforms are shown as dashed gray lines resulting in products 1–5. For splice isoforms 1–3, the longest ORF is 1 amino-acid (aa) as shown by the start-stop codons, whereas isoforms 4 and 5 could encode proteins of 57 aa and 33 aa in length, respectively. Predicted splice sites are supported by RNA-seq data (SRR3955476).
(PDF)

S4 Fig Sequence of intron/riboswitch constructs inserted into RFP. (PDF)

S5 Fig Cladogram showing all proteins and species names from Fig 3A (*DUR3/DUR31* phylogeny). Bootstrap values (out of 100) are shown for each branch point.
(PDF)

S1 Data Set Location of open reading frames and riboswitches. (XLSX)

S1 Table List of primers used. (DOCX)

S2 Table List of strains used. (DOCX)
