# Supplementary material for: TPP riboswitch-dependent regulation of an ancient thiamin transporter in Candida
Source: PLoS Genet. 2018 May 31;14(5):e1007429. doi: 10.1371/journal.pgen.1007429 (PMC5997356; doi:10.1371/journal.pgen.1007429)
Supplement: S1 Fig — 16 Thi7 homologs from Saccharomycotina species were used to generate a Thi7 HMM, which was used with HMMER to search all species from Fig 5. The amino acid sequences of predicted Thi7 homologs were aligned using Muscle (v3.8.31), and a phylogenetic tree was constructed using RAxML. Thi7 is amplified in S. cerevisiae as shown (Thi7, Thi72, Nrt1). The three related S. cerevisiae proteins Fui1, Dal4, and Fur4 are also shown. The arrow shows the most-likely cut-off for Thi7 orthologs. The species are colored using the format in Fig 5 (Saccharomycetaceae (dark blue), Saccharomycodaceae (sea green), Phaffomycetaceae (cyan), Yarrowia (red), Debaryomycetaceae/Metschnikowiaceae (yellow), Pichiaceae (orange), Pezizomycotina (gray). (PDF) [file pgen.1007429.s001.pdf]

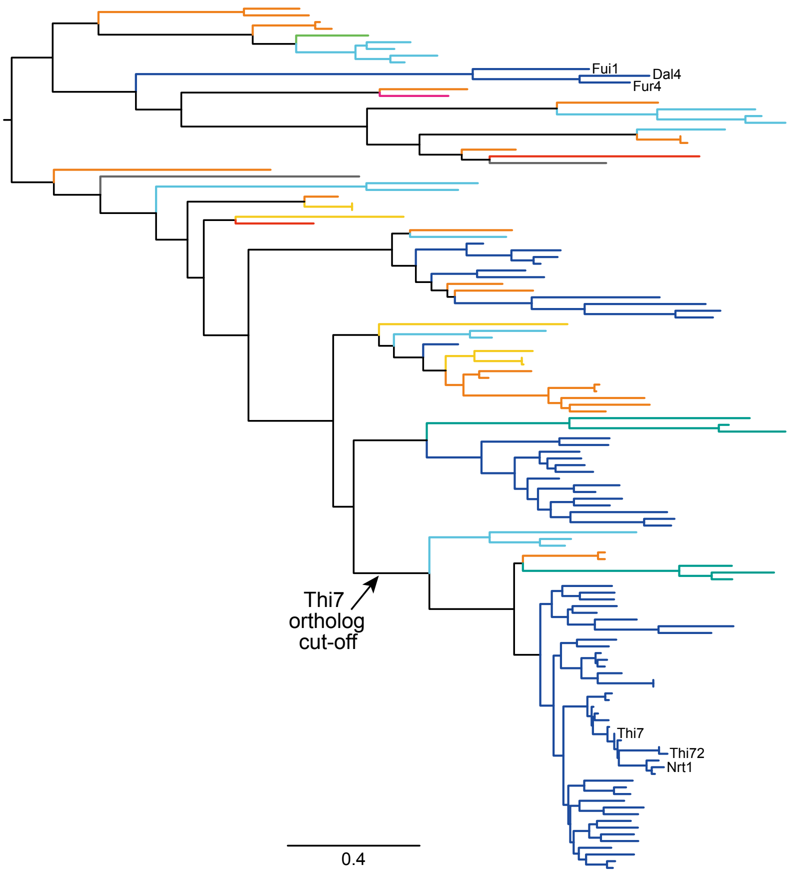

S1 Figure. Phylogeny of Thi7 and related proteins from Saccharomycotina species.

16 Thi7 homologs from Saccharomycotina species were used to generate a Thi7 HMM, which was used with HMMER to search all species from Fig. 5. The amino acid sequences of predicted Thi7 homologs were aligned using Muscle (v3.8.31), and a phylogenetic tree was constructed using RAxML. Thi7 is amplified in *S. cerevisiae* as shown (Thi7, Thi72, Nrt1). The three related *S. cerevisiae* proteins Fui1, Dal4, and Fur4 are also shown. The arrow shows the most-likely cut-off for Thi7 orthologs. The species are colored using the format in Fig. 5 (Saccharomycetaceae (dark blue), Saccharomycodaceae (sea green), Phaffomycetaceae (cyan), Yarrowia (red), Debaryomycetaceae/Metschnikowiaceae (yellow), Pichiaceae (orange), Pezizomycotina (gray).
