# Supplementary material for: TPP riboswitch-dependent regulation of an ancient thiamin transporter in Candida
Source: PLoS Genet. 2018 May 31;14(5):e1007429. doi: 10.1371/journal.pgen.1007429 (PMC5997356; doi:10.1371/journal.pgen.1007429)
Supplement: S2 Fig — The alignment was generated using T-Coffee [53] and visualized using SeaView [54]. The predicted riboswitch secondary structure is shown below, where ‘><‘ symbols indicate potential complementary nucleotides, ‘P1/P2’ shows the riboswitch stems, and ‘ …’ depicts loops and other unmatched nucleotides. Stem P3 is highly variable, as previously reported [7]. (PDF) [file pgen.1007429.s002.pdf]

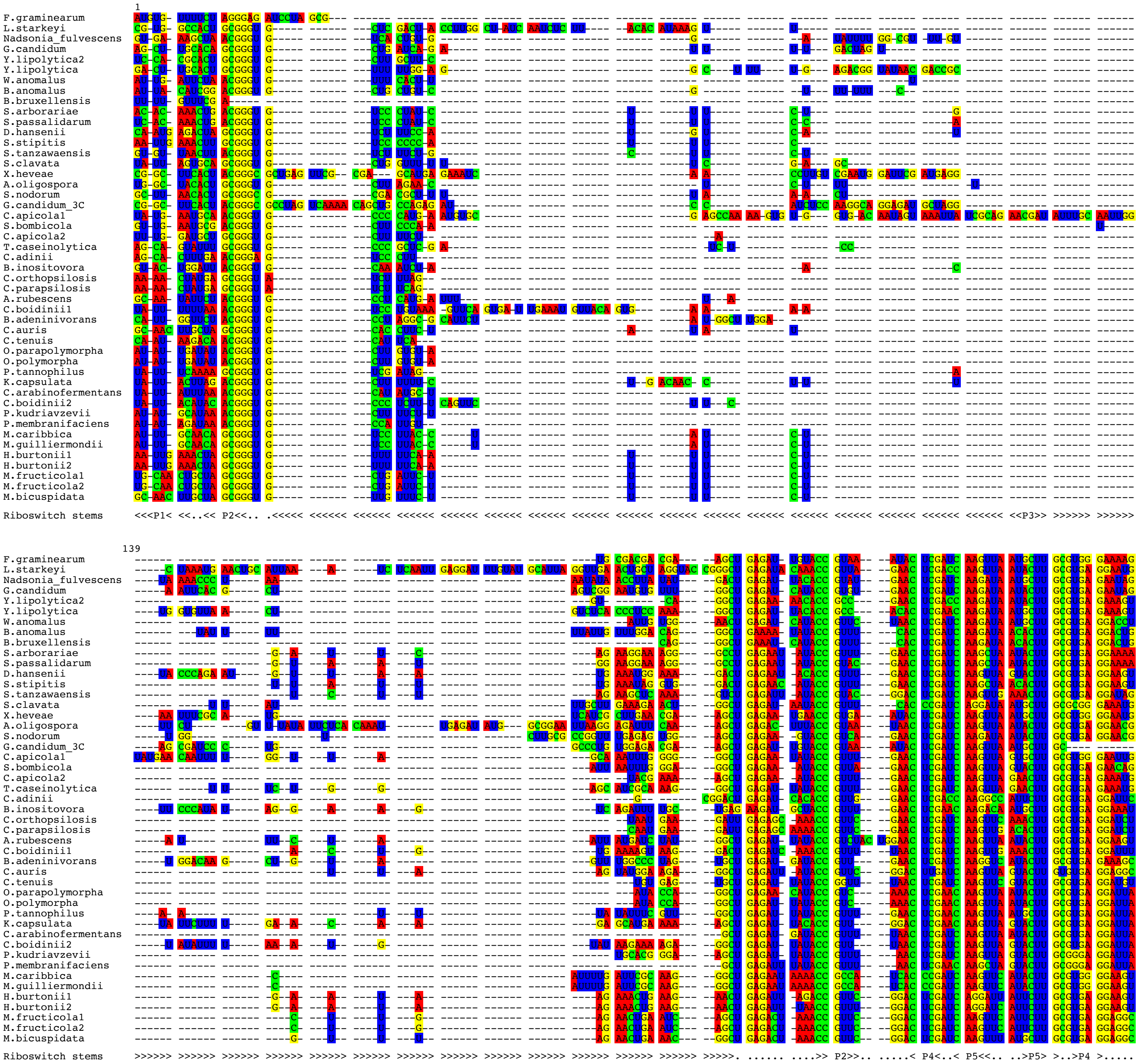

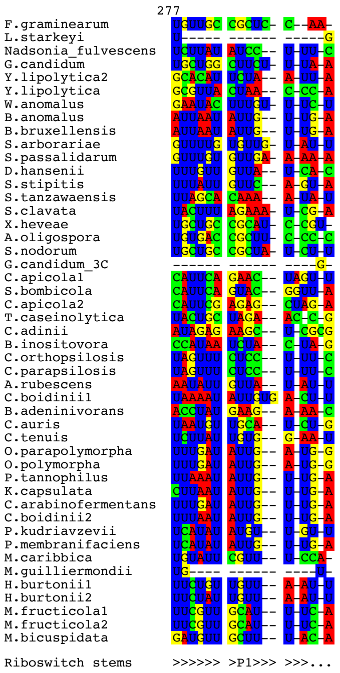

S2 Figure. Alignment of all riboswitches predicted in *DUR31* genes from the species shown in Fig. 1. The alignment was generated using T-Coffee [53] and visualized using SeaView [54]. The predicted riboswitch secondary structure is shown below, where '><' symbols indicate potential complementary nucleotides, 'P1/P2' shows the riboswitch stems, and '...' depicts loops and other unmatched nucleotides. Stem P3 is highly variable, as previously reported [7].
