# Supplementary material for: TPP riboswitch-dependent regulation of an ancient thiamin transporter in Candida
Source: PLoS Genet. 2018 May 31;14(5):e1007429. doi: 10.1371/journal.pgen.1007429 (PMC5997356; doi:10.1371/journal.pgen.1007429)
Supplement: S3 Fig — The gray, cyan, and magenta bars represent genomic DNA. The gray region depicts DNA outside the TPP riboswitch intron, the cyan depicts DNA inside the intron, and the magenta region depicts the TPP riboswitch. Potential splice isoforms are shown as dashed gray lines resulting in products 1–5. For splice isoforms 1–3, the longest ORF is 1 amino-acid (aa) as shown by the start-stop codons, whereas isoforms 4 and 5 could encode proteins of 57 aa and 33 aa in length, respectively. Predicted splice sites are supported by RNA-seq data (SRR3955476). (PDF) [file pgen.1007429.s003.pdf]

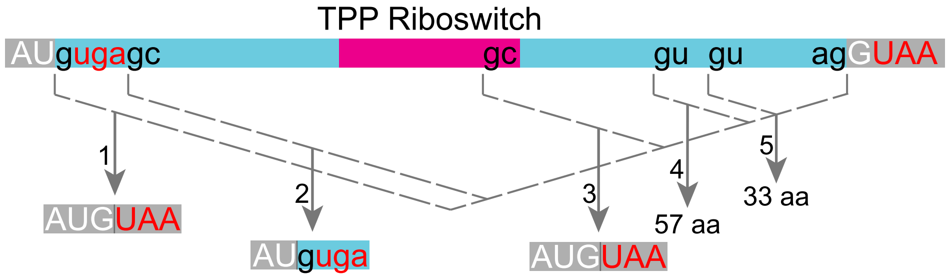

Fig. S3. The TPP riboswitch in *Brettanomyces bruxellensis* is in a region with poor protein-coding potential.

The grey, cyan, and magenta bars represent genomic DNA. The grey region depicts DNA outside the TPP riboswitch intron, the cyan depicts DNA inside the intron, and the magenta region depicts the TPP riboswitch. Potential splice isoforms are shown as dashed grey lines resulting in products 1 - 5. For splice isoforms 1 - 3, the longest ORF is 1 amino-acid (aa) as shown by the start-stop codons, whereas isoforms 4 and 5 could encode proteins of 57 aa and 33 aa in length, respectively. Predicted splice sites are supported by RNA-seq data (SRR3955476).
