# Supplementary material for: TPP riboswitch-dependent regulation of an ancient thiamin transporter in Candida
Source: PLoS Genet. 2018 May 31;14(5):e1007429. doi: 10.1371/journal.pgen.1007429 (PMC5997356; doi:10.1371/journal.pgen.1007429)
Supplement: S4 Fig — (PDF) [file pgen.1007429.s004.pdf]

Fig. S4. Squence of G blocks used to insert riboswitch in RFP.

5’ and 3’ splice sites
Riboswitch

>*Candida parapsilosis CPAR2_502100* intron with riboswitch (Integrated DNA Technologies), 351 bp
GTATGTGAAAGAGAACCACATCACACAGAAAGAATGAGAATAGCACTGA
GGAATCCTAATCTAAAAATCGAAAAGCAAAGGTTGGACACACAAACCATT
GTGGAGGACGACTATCTCCACCACACTGAAACGGAAAAACTATGAGCGG
GTATCTTCAGCAATGAAGATTGAGAGCAAAACCGTTCGAACTCGATCAAG
TTGACACTTGCGTGAGGATCTTAGTTTCTCCTTTCTTGAAAATAACAAAGC
AAAGTGGGGTGGTGGCGGAAAGATGCCGACCCTTTGAATAAGACTGCAC
TACCACCACCCACTTTGTCCCATTTCAATTTGTCAATACTAACTTGGTACT
AG

>*Candida parapsilosis CPAR2_502100* intron without riboswitch (Integrated DNA Technologies), 252 bp
GTATGTGAAAGAGAACCACATCACACAGAAAGAATGAGAATAGCACTGA
GGAATCCTAATCTAAAAATCGAAAAGCAAAGGTTGGACACACAAACCATT
GTGGAGGACGACTATCTCCACCACACTGAAACGGATTGAAAATAACAAA
GCAAAGTGGGGTGGTGGCGGAAAGATGCCGACCCTTTGAATAAGACTG
CACTACCACCACCCACTTTGTCCCATTTCAATTTGTCAATACTAACTTGGT
ACTAG
