# Supplementary material for: TPP riboswitch-dependent regulation of an ancient thiamin transporter in Candida
Source: PLoS Genet. 2018 May 31;14(5):e1007429. doi: 10.1371/journal.pgen.1007429 (PMC5997356; doi:10.1371/journal.pgen.1007429)
Supplement: S5 Fig — Bootstrap values (out of 100) are shown for each branch point. (PDF) [file pgen.1007429.s005.pdf]

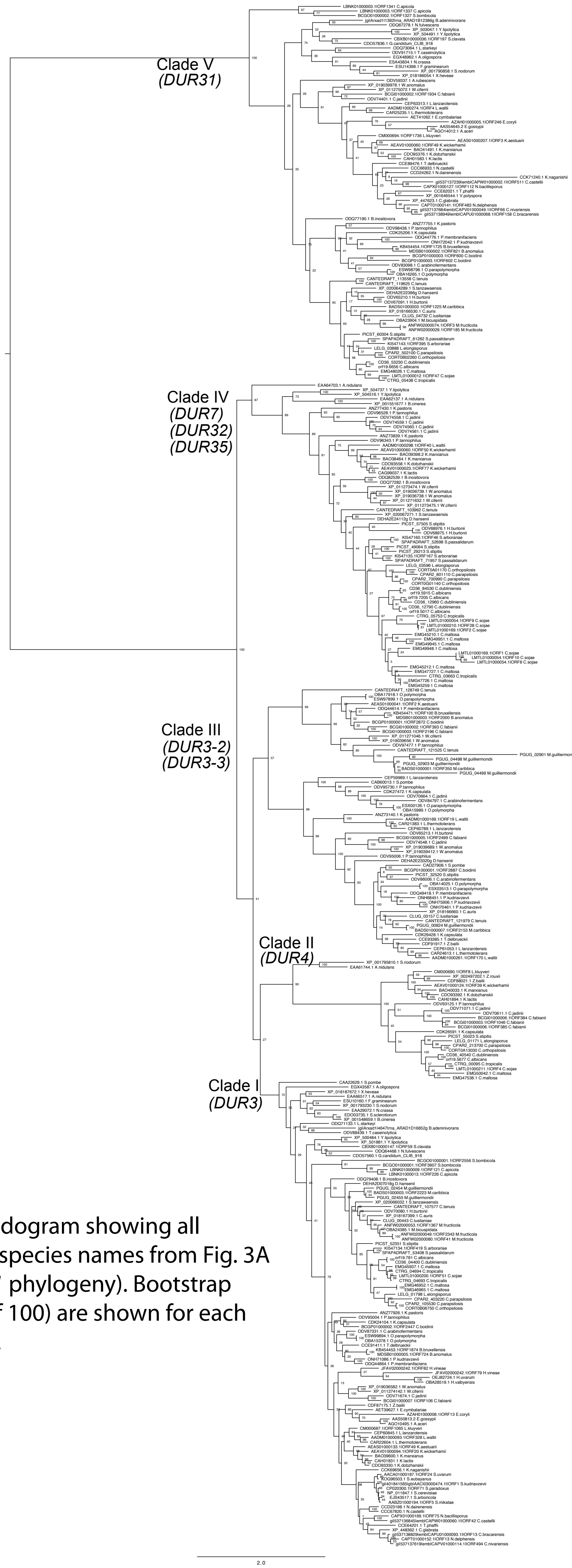

S5 Figure. Cladogram showing all proteins and species names from Fig. 3A (*DUR3/DUR31* phylogeny). Bootstrap values (out of 100) are shown for each branch point.
