# Supplementary material for: TPP riboswitch-dependent regulation of an ancient thiamin transporter in Candida
Source: PLoS Genet. 2018 May 31;14(5):e1007429. doi: 10.1371/journal.pgen.1007429 (PMC5997356; doi:10.1371/journal.pgen.1007429)
Supplement: S1 Table — (DOCX) [file pgen.1007429.s007.docx]

**S1 Table.** Strains used in this study.

| **Species** | **Strain** | **Genotype** | **Source** |
| --- | --- | --- | --- |
| *E. coli* | DH5α | F– endA1 glnV44 thi-1 recA1 relA1 gyrA96 deoR nupG purB20 φ80dlacZΔM15 Δ(lacZYA-argF)U169, hsdR17(rK–mK+), λ– | Common laboratory strain |
| *S. cerevisiae* | BY4741 | MATa; ura3Δ0; leu2Δ0; his3Δ1; met15Δ0 | ^51^ |
| *C. albicans* | SC5314 | Wildtype strain | ^52^ |
| *C. albicans* | M1614 | orf19.6656Δ::ARG4/orf19.6656Δ::HIS1 rps10::URA3 | ^20^ |
| *C. albicans* | M1408 | orf19.6656Δ::ARG4/orf19.6656Δ::HIS1 rps10::URA3 | ^20^ |
| *C. albicans* | CAF 4-2 | Δura3::imm434/ura3::imm434 | ^19^ |
| *C. albicans* | dur3Δ | Δura3::imm434/Δura3::imm434, Δdur3::FRT | ^19^ |
| *C. albicans* | dur31Δ | Δura3::imm434/Δura3::imm434, Δdur31::FRT | ^19^ |
| *C. albicans* | dur3Δ/DUR3 | Δura3::imm434/Δura3::imm434, dur3/DUR3::FRT/RP10::DUR3ORF | ^19^ |
| *C. albicans* | dur3Δ/dur31Δ | Δura3::imm434/Δura3::imm434, Δdur3::FRT/Δdur31::FRT | ^19^ |
| *C. albicans* | DUR3 O/E | Δura3::imm434/Δura3::imm434, DUR3/DUR3:FRT RP10::DUR3ORF | ^19^ |
| *C. parapsilosis* | CLIB214 | Wildtype strain | Type strain |
| *C. parapsilosis* | CPL2H1 | leu2::FRT/leu2::FRT, his1::FRT/his1::FRT | ^53^ |
| *C. parapsilosis* | dur3Δ | leu2::FRT/leu2::FRT, his1::FRT/his1::FRT, dur3::LEU2/ dur3::HIS1 (CPL2H1 background) | This work |
| *C. parapsilosis* | dur31Δ | leu2::FRT/leu2::FRT, his1::FRT/his1::FRT, dur31::LEU2/ dur31::HIS1 (CPL2H1 background) | This work |
