# Supplementary material for: TPP riboswitch-dependent regulation of an ancient thiamin transporter in Candida
Source: PLoS Genet. 2018 May 31;14(5):e1007429. doi: 10.1371/journal.pgen.1007429 (PMC5997356; doi:10.1371/journal.pgen.1007429)
Supplement: S2 Table — (DOCX) [file pgen.1007429.s008.docx]

**S2 Table** Oligonucleotide primers

| **Oligo name** | **Sequence (5’ - 3’)** |
| --- | --- |
| ***C. parapsilosis DUR31* deletion** | |
| CpDUR31_1 | CATTATTTACCTCACATATA |
| CpDUR31_2* | ccgctgctaggcgcgccgtgACCAGTGTGATGGATATCTGC |
| CpDUR31_3* | cacggcgcgcctagcagcggAGCTAGTACCAAGTTAGTAT |
| CpDUR31_4* | gtcagcggccgcatccctgcATATTTTTAGATTATGCAGT |
| CpDUR31_5* | gcagggatgcggccgctgacGTGCAACTGAAACGCAGCCAagctcggatccactagtaacg |
| CpDUR31_6 | AATTAACCCACATACTTTAG |
| CpDUR31_5check | ATTTGTTTCAAGGCGGAAAA |
| CpDUR31_3check | TAGACGCATCAAAGGCACCA |
| CpDUR31_ORF_F | GGTTAAATGCATTGGGTGCT |
| CpDUR31_ORF_R | GCCCAATTGGTGTGAAGTTT |
| ***C. parapsilosis DUR3* deletion** | |
| CpDUR3_1 | ACATACATCAAATTTTGGCC |
| CpDUR3_2* | ccgctgctaggcgcgccgtgACCAGTGTGATGGATATCTGC |
| CpDUR3_3* | cacggcgcgcctagcagcggTGGGTGATATGTATAGTGTA |
| CpDUR3_4* | gtcagcggccgcatccctgcCTTCTTTAGATGTTCTTCAG |
| CpDUR3_5* | gcagggatgcggccgctgacAGATGTCGTCAACCTGAGCCagctcggatccactagtaacg |
| CpDUR3_6 | ATAGATAAGGCACAGACTAA |
| CpDUR3_5check | AATTGCACATAAAAATTTAT |
| CpDUR3_3check | TCCTTGCATGTACTGTTCAT |
| CpDUR3_ORF_F | GTGGCCGCTTGTTTATTGAT |
| CpDUR3_ORF_R | CAATTCGGATGACATTGCAC |
| **Analysis of alternative splicing in *O. polymorpha*** | |
| HpDUR31f1 | TGGTACAAGGACGGGTCTTC |
| HpDUR31f2 | TTTCAAGCAGCGAATGCCATC |
| HpDUR31r1 | GAGAGAACAGGAAGATAGGAATGG |
| HpDUR31r2 | TGAATGCCGACAGATACAAAGC |
| **Analysis of alternative splicing in *C. parapsilosis*** | |
| CP_TPP_F | AGCACCCAATGCATTTAACC |
| CP_TPP_F2 | TCTATGCCTTACCCACTCGG |
| CP_TPP_R1 | GAGCCCATCTCATCTTTCCA |
| CP_TPP_R2 | GAAAGAGAACCACATCACACA |
| CP_TPP_R3 | TGCCTCTAAGACCGATTTCC |
| **Amplification of pRS316-GAP-yEmRFP** | |
| RFP_Lin-1_F | GAATTTATGAGATTTAAAGTTCAT |
| RFP_Lin-1_R | TTTAATAATAGCCATATTATCTTCTTC |
| **Amplification of *C. parapsilosis* *DUR31* intron and TPP riboswitch Gblocks** | |
| Cpi-1_F* | ataatatggctattattaaaGTATGTGAAAGAGAACCACATC |
| Cpi-1_R* | actttaaatctcataaattcCTAGTACCAAGTTAGTATTGAC |
| **Primers for the detection of alternative splicing in *S. cerevisiae* BY4741 +plasmids** | |
| RFPcheck_F | AGGTGAAGAAGATAATATGGC |
| GapRFP_R | CCATACATAAATTGTGGTGAC |

* lowercase letters indicate complementary sequences
